# Perinatal S100B Protein Assessment in Human Unconventional Biological Fluids: A Minireview and New Perspectives

**DOI:** 10.1155/2010/703563

**Published:** 2010-06-16

**Authors:** Diego Gazzolo, Fabrizio Michetti

**Affiliations:** ^1^Department of Maternal, Fetal and Neonatal Medicine, C. Arrigo Children's Hospital, Alessandria, Italy; ^2^Research Laboratory, Department of Cardiac Surgery, San Donato University Hospital, San Donato Milanese, Italy; ^3^Institute of Anatomy and Cell Biology, Catholic University of the Sacred Heart, Largo Francesco Vito, 1, I-00168 Rome, Italy

## Abstract

Growing evidence is now available on the use of S100B protein as a valuable marker of brain damage and its role as a neurotrophic factor. Bearing in mind, among different S100B protein properties that are still being investigated, the possibility of measuring this protein in different biological fluids renders it suitable for use in several disciplines. This is the case with perinatal medicine where even more noninvasive techniques are particularly desirable in order to ensure the minimal handling diagnostic and therapeutic strategies. In this setting, the present minireview reports data on the presence and the usefulness of S100B protein as brain damage marker and as a neurotrophic factor in the so-called unconventional biological fluids such as saliva and human milk, respectively. Results offer new possibilities for the use of S100B in perinatal medicine as a key-protein for the investigations focusing on central nervous system development and damage.

## 1. Introduction

The term S100B refers to a member of a multigenic family of calcium-modulated proteins first identified in 1965 by Moore [1], each of which exhibits a unique pattern of tissue- or cell type-specific expression; the term S100 refers to their solubility in a 100%-saturated solution with ammonium sulfate. To date at least 25 proteins have been identified as belonging to the S100 protein family, characterized by the presence of a pair of so-called EF-hand (i.e., helix-loop-helix) calcium-binding motifs, first discovered in the crystal structure of parvalbumin, that induce conformational changes of the protein after binding to calcium. Most S100 proteins exist as dimers (frequently homodimers) within cells and are generally expressed and distributed in a cell-definite fashion, indicating a conserved biological role. In this setting, S100B is a homodimer of the beta subunit, mainly concentrated in the nervous system and in the adipose tissue. In the nervous system S100B appears to be most abundant in glial cells and its presence in specific neuronal subpopulations has also been described [2–6]. S100B is present intracellularly and extracellularly; it is believed to regulate several cellular functions (protein phosphorylation, protein degradation, calcium homeostasis, cell locomotion, transcription factors, cell proliferation and differentiation, enzymes, regulation of receptor function, cytoskeleton) while its biological intracellular role has not yet been completely elucidated in unifying terms. An extracellular biological role is also attributed to S100B, which is secreted by astrocytes as a cytokine exerting an autocrine or paracrine effect on glia, neurons and microglia; the protein may have a trophic effect during both development and nerve regeneration at physiologic (nanomolar) concentrations (the Jekyll side), but at high (micromolar) concentrations (the Hyde side) could be neurotoxic, participating in the pathophysiology of neurodegenerative disorders [7–10]. In this respect, transcriptional effects of micromolar S100B on neuroblastoma cells have been shown to result in perturbation of cholesterol homeostasis and interference in the cell cycle [11]. Both effects of S100B on target cells are believed to be mediated through RAGE engagement [5]. Apart from its still unknown function, the presence of S100B in biological fluids is interesting, since at present it constitutes an established index of brain injury [10]. It should be noted in this respect that its half-life is approximately 1 hour and it is mainly eliminated by the kidney [12]. In particular, the assessment of S100B in biological fluids has been usefully employed in perinatal medicine [12, 13]. After its established use in conventional fluids (CSF, blood, urine, amniotic fluid), this minireview is focused on the assessment of S100B levels in unconventional fluids (saliva, milk), which may open new perspectives for studies on this protein.

### 1.1. S100B and Conventional Biological Fluids

S100B protein has been measured in several biological fluids (cerebrospinal fluid, blood, urine and amniotic fluid) by a series of immunoassays, which have been variously used in different fluids. 

Cerebrospinal fluid (CSF)was the first of various biological fluids in which the role of S100B as a marker of active brain damage was demonstrated [14, 15]. In perinatal medicine, measurements of S100B protein in CSF have been used to monitor infants affected by perinatal asphyxia and post-hemorrhagic ventricular dilatation brain damage during cardiac surgery. S100B concentrations correlated with the extent of brain lesions, with long-term prognosis, and with neurological impairment at 1 year of age or death before that time [16–18].

The idea of measuring S100B into blood was based on the hypothesis that during active brain injury at least some of the S100B released from the damaged tissue could spread into the systemic circulation [19], also as a result of hemodynamic rearrangement of the blood brain barrier. Increased blood concentrations of S100B were indeed detected in cases of chronic hypoxia and/or intraventricular hemorrhage (IVH) in preterm infants, in full-term infants suffering by perinatal asphyxia and adverse neurological outcomes [20–23]. S100B was also measured in the blood of women whose pregnancies are complicated by intrauterine growth retard and whose newborns develop intraventricular haemorrhage [24].

In the urine S100B concentrations at birth were significantly higher in preterm newborns developing intraventricular haemorrhage and/or brain damage. In a cross-sectional study using urine obtained from preterm newborns, the protein has been found to be a valuable predictor of early neonatal death [25–28].

In the amniotic fluid S100B concentration has been shown to correlate with gestational age and with cerebral ultrasound scanning results in healthy fetuses [29]. In addition, amniotic S100B concentrations are higher in monoamniotic than in diamniotic and singleton pregnancies, hinting at the possibility that each foetus releases a physiologically defined amount of S100B during pregnancy [30]. Elevated S100B concentrations in the amniotic fluid have also been shown to constitute a reliable marker of foetus pathological conditions, including trisomy 21 [31–35].

### 1.2. S100B and Unconventional Biological Fluids

Among human biological fluids, in the perinatal period, the studies aimed at investigating the presence of S100B protein in biological fluids, namely “unconventional” biological fluids such as saliva and milk, are especially intriguing. In particular saliva, which is more easily collected than CSF, blood or urine offers the hope of simple non-invasive tests especially useful in perinatal medicine. This possibility could even represent the reaching of the so called “gold standard” for non-invasive longitudinal monitoring of occurring brain damage eagerly awaited in clinical practice. Future prospects also include the possibility of monitoring the true effectiveness of the even more risky therapeutic strategies such as mechanical ventilation, brain cooling and experimental medications. Even more interesting prospects are offered by the detection of S100B in breast milk, candidating the protein as a participant in the biochemical communication between mother and child. This might contribute to the identification of biochemical markers with a series of unknown functions that to date confirm that the role of maternal milk instead of artificial ones in brain maturation involvement is unique.

#### 1.2.1. S100B and Saliva

The possibility that S100B could be assessed in different biological fluids has been substantiated in the last decade, thanks to continuous interactions among physicians of several disciplines. In this regard, the concept of minimal handling of patients derives from neonatologists and perinatologists who need, in the phase of critical intensive care medicine, non-invasive parameters able to detect cases at risk of brain damage at the earliest stage in order to prevent it. Among non-invasive biological fluids saliva meets the optimality requirements since it can be collected any time without newborn stress, its measurements are reproducible and can be compared with other “invasive” biological fluids.

Studies of human fetuses have shown that members of the S100 protein family are present in different tissues of the salivary glands during ontogenesis. Results showed that different S100 proteins are expressed during different phases of pregnancy up to adulthood, whilst S100B in particular has been found to be essentially absent from fetal salivary glands from 32 weeks of gestation onwards [36, 37]. Data on physiology of salivary glands reported that systemic blood and saliva are isotonic and contain the same concentrations of ions in the same ratios, thanks to a continuous exchange of ions and protein between blood and saliva. In addition, an increase or a decrease in the volume of saliva release is systemic circulation-dependent and mediated by ortho/parasympathetic activation [38]. These findings indicate that the salivary glands do not produce S100B in the third trimester of pregnancy and that S100B concentrations detected in saliva likely derive from systemic circulation. The first observation of the presence of S100B in saliva was supported by immunoluminometric assay confirmed by Western blot analysis and also offered a reference curve of the protein in this biological fluid in normal preterm and term newborns [39]. Results showed that saliva can be used in the clinical monitoring of S100B levels, which, as already established for other biological fluids, such as CSF, blood, urine or amniotic fluid, can constitute a reliable index to diagnose/monitor brain distress. Since saliva samples are more easily collected than other biological fluids, tests performed on this biological fluid appear to be especially convenient in the clinical evaluation of newborns.

#### 1.2.2. S100B and Milk

The hypothesis of assessing the protein in a so complex biological fluid was based on the following elements: (i) S100 proteins are highly conserved in amino acid composition among vertebrate species, suggesting a pivotal, although still unknown, biological role for the protein [40]; (ii) S100-like proteins have also been immunologically detected in spinach leaves suggesting a potential role in human food-chain [41], adipose tissue also constitutes a site of concentration for the protein [42]; (iii) breast milk is known to contain growth factors and cytokines [43–46]; (iv) milk is abundant in other calcium-binding proteins (e.g., alpha-lactalbumin, calmodulin, osteocalcin) [47–49]. Results showed S100B in human breast milk; in addition, S100B milk content was estimated to be 80/100 times higher than those detected in CSF, blood, urine [50]. Interestingly, significant differences have been found in S100B milk concentrations among different mammalian species such as human, cow, goat, donkey and sheeps. The S100B concentration is higher in human milk, thus raising possible speculations on a relationship between the milk concentration of this neurotrophic factor and species evolution [51]. Western blot analysis confirmed that the immunoreactivity observed using immunoluminometric assay refers to S100B protein and RT-PCR analysis also detected human S100B mRNA in the human milk [50]. This latter finding likely refers to the presence in the milk of cell types expressing S100B, including mammary epithelial cells and lymphocytes, which reasonably may be supposed to be the source of S100B in the milk. The presence of S100B at very high concentration in human breast milk may be related to its putative neurotrophic role, given that breast-feeding is believed to exert a stimulatory effect on brain maturation [52]. More detailed information will be needed to corroborate the possibility that S100B may participate in the nutritional aspects of milk, including possible effects on intestinal development and/or trophism of the enteric nervous system. In this respect it should be noted that human breast milk is known to contain different substances that may actively influence infant growth and development, including hormones, growth factors and cytokines [53]. Interestingly, S100B levels in human breast milk significantly increase during milk maturation, being very low in colostrum, intermediate in transition milk (7 and 14 days) and high in mature milk (30 days) [54]. Further studies investigated the impact of industrial preparation procedures (skimmed cow milk, protein sources supplementation, pasteurization and spray-drying procedures) on S100B content. Results showed that S100B has a sufficient thermo-stability to resist pasteurization but not spry-drying, suggesting that new feeding strategies in preterm and term infants are therefore warranted in order to preserve S100B and other potential brain constituents during industrial preparation [55]. It may be relevant in this respect that, thanks to the considerable technological improvement in dietetics preparations and progresses in laboratory biochemistry, evidence is growing that artificial milks could need to be enriched with recent discovered constituents: S100B can be included among these on account of its neurotrophic properties.

Finally, apart from the possible trophic role on the newborn exerted by the maternal breast milk, the examination of S100B level in this unconventional biological fluid could offer new prospects in the investigation of maternal physiopathological conditions with a potential direct impact on the newborn health.
